# Psychological impact of risk-stratified screening as part of the NHS Breast Screening Programme: multi-site non-randomised comparison of BC-Predict versus usual screening (NCT04359420)

**DOI:** 10.1038/s41416-023-02156-7

**Published:** 2023-02-11

**Authors:** David P. French, Lorna McWilliams, Sarah Bowers, Victoria G. Woof, Fiona Harrison, Helen Ruane, Alice Hendy, D. Gareth Evans

**Affiliations:** 1 Manchester Centre of Health Psychology, Division of Psychology and Mental Health, School of Health Sciences, University of Manchester, Coupland Street, Manchester, M13 9PL England; 2 NIHR Manchester Biomedical Research Centre, Manchester Academic Health Science Centre, Central Manchester University Hospitals NHS Foundation Trust, Manchester, England; 3 Manchester Breast Centre, Manchester Cancer Research Centre, University of Manchester, 555 Wilmslow Rd, Manchester, M20 4GJ England; 4 The Nightingale and Prevent Breast Cancer Centre, Manchester University NHS Foundation Trust, Manchester, M23 9LT England; 5 Genomic Medicine, Division of Evolution and Genomic Sciences, The University of Manchester, St Mary’s Hospital, Manchester University NHS Foundation Trust, Manchester, M13 9WL England

**Keywords:** Preventive medicine, Human behaviour, Predictive markers

## Abstract

**Background:**

Adding risk stratification to standard screening via the NHS Breast Screening Programme (NHSBSP) allows women at higher risk to be offered additional prevention and screening options. It may, however, introduce new harms such as increasing cancer worry. The present study aimed to assess whether there were differences in self-reported harms and benefits between women offered risk stratification (BC-Predict) compared to women offered standard NHSBSP, controlling for baseline values.

**Methods:**

As part of the larger PROCAS2 study (NCT04359420), 5901 women were offered standard NHSBSP or BC-Predict at the invitation to NHSBSP. Women who took up BC-Predict received 10-year risk estimates: “high” (≥8%), “above average (moderate)” (5–7.99%), “average” (2–4.99%) or “below average (low)” (<2%) risk. A subset of 662 women completed questionnaires at baseline and at 3 months (*n* = 511) and 6 months (*n* = 473).

**Results:**

State anxiety and cancer worry scores were low with no differences between women offered BC-Predict or NHSBSP. Women offered BC-Predict and informed of being at higher risk reported higher risk perceptions and cancer worry than other women, but without reaching clinical levels.

**Conclusions:**

Concerns that risk-stratified screening will produce harm due to increases in general anxiety or cancer worry are unfounded, even for women informed that they are at high risk.

## Introduction

National screening programmes for breast cancer such as the National Health Service Breast Screening Programme (NHSBSP) aim to detect and treat breast cancers earlier and thereby reduce mortality [1]. However, all screening programmes cause harms notably false-positive screening test results and overdiagnosis. Although major reviews have concluded that the benefits of breast cancer screening, mainly in terms of lives saved, outweigh the harms [2], it is important to identify any innovations which improve the ratio of benefits to harms.

One innovation to improve the ratio of benefits to harms is to risk-stratify screening, whereby women with different levels of risk are offered different detection and prevention options [3]. Notably, the National Institute for Health and Care Excellence (NICE) recommended that women at high risk of breast cancer should be offered more frequent screening by mammography (annual between 40 and 60 years) and risk-reducing medication (tamoxifen or aromatase inhibitors) to reduce risk of cancer [4]. However, these NICE guidelines cannot currently be implemented with the majority of women who might benefit from these detection and prevention options, as their risk status is unknown [5, 6] Assessing risk status at screening would allow this to happen, and trials are underway internationally to establish effectiveness in terms of reducing the number of advanced (stage 2+) breast cancers [7, 8].

Before a service that offers risk-stratified screening could be implemented, it is important to establish whether risk-stratified screening would induce harms and if so, how they can be mitigated so as not to outweigh benefits [9]. A major potential harm of risk-stratified screening is that providing women with information about their risk of breast cancer will cause high levels of worry about cancer, or high general levels of anxiety [10]. This was recently examined in women participating in the Predicting-Risk-Of-Cancer-At-Screening (PROCAS) study [11] where risk estimates were produced based on the Tyrer–Cuzick model incorporating up to three sources of information: (a) self-reports e.g., of family history, parity, BMI, height, age at menarche/menopause/at first live birth, HRT use, (b) breast density, obtained from mammography, and (c) genetic information, i.e., single-nucleotide polymorphisms (SNPs) derived from saliva [12].

The PROCAS study found no evidence of major harm from receiving risk estimates. The women who received risk estimates reported slightly higher cancer worry scores, but lower general anxiety scores than comparison women awaiting results [13]. Scores on both variables were low, and general anxiety scores were lower (mean scores of 10.4) than those found in previous research in England with women invited to breast cancer screening which found a mean general anxiety score of 11.1 [14]. These findings are particularly notable as it also found that women’s perceptions of risk changed in line with the risk estimates they were given, suggesting that the information given was understood [13].

This analysis had several limitations, however. First, in the PROCAS study, communication of risk estimates happened approximately three years after women provided their questionnaire data and consent, as the main purpose of that study was to validate risk prediction algorithms rather than assess a new screening service model [11]. Second, the study did not include women at the highest levels of risk (i.e., 8% or higher risk over 10 years), as they had already been identified and offered additional screening and cancer-preventing medication shortly after providing information about their risk status [15]. Third, the comparison group was women participating in PROCAS who were awaiting their risk results, and who may have had elevated anxiety or cancer worry themselves. Finally, no baseline measures of anxiety or cancer worry were taken for any women in this study, so it was not possible to identify changes in these variables, and thereby attribute changes to the receipt of particular pieces of information.

There are also potential benefits of risk-stratified screening including the receipt of personalised risk information promoting behaviour change that could reduce the risk of breast cancer. The best evidence to date on this point from the PROCAS study does not suggest this is likely, but, the evidence base has a number of limitations as previously articulated [11]. A further potential benefit is increased knowledge to make an informed personal decision about whether to attend screening, and any treatment options that follow from screening [16]. There is currently an absence of evidence on this point [10].

The overall aim of the present research was therefore to establish whether providing women eligible for NHSBSP with BC-Predict increases potential harms (notably anxiety and cancer worry) and benefits (notably knowledge and intentions to attend future screening), at 3 months and 6 months post screening. Specific objectives were as follows, in line with a pre-specified research protocol:

1. Are there differences in self-report measures of harms and benefits between women offered BC-Predict compared to women offered NHSBSP, controlling for baseline values?
2. Are there differences in self-report measures of harms and benefits between women who accept BC-Predict compared to women who decline BC-Predict, controlling for baseline values?
3. Are there differential changes in self-report measures of harms and benefits for the four groups of women provided with different risk estimates (i.e., high, moderate, average and below average) by BC-Predict?

## Materials and methods

### Design

The present research was a nested study embedded within the larger PROCAS2 study [17]. PROCAS2 was a non-randomised controlled trial of the effects of offering women either standard NHSBSP or BC-Predict as part of the NHSBSP offer. NHS ethical approval for the larger PROCAS2 study was granted by Harrow Research Ethics Committee (ref 18/LO/0649)/ IRAS project ID 239199 and this nested questionnaire study by North West—Greater Manchester East Research Ethics Committee (ref 18/NW/0856)/IRAS project ID 248052.

**Fig. 1:**
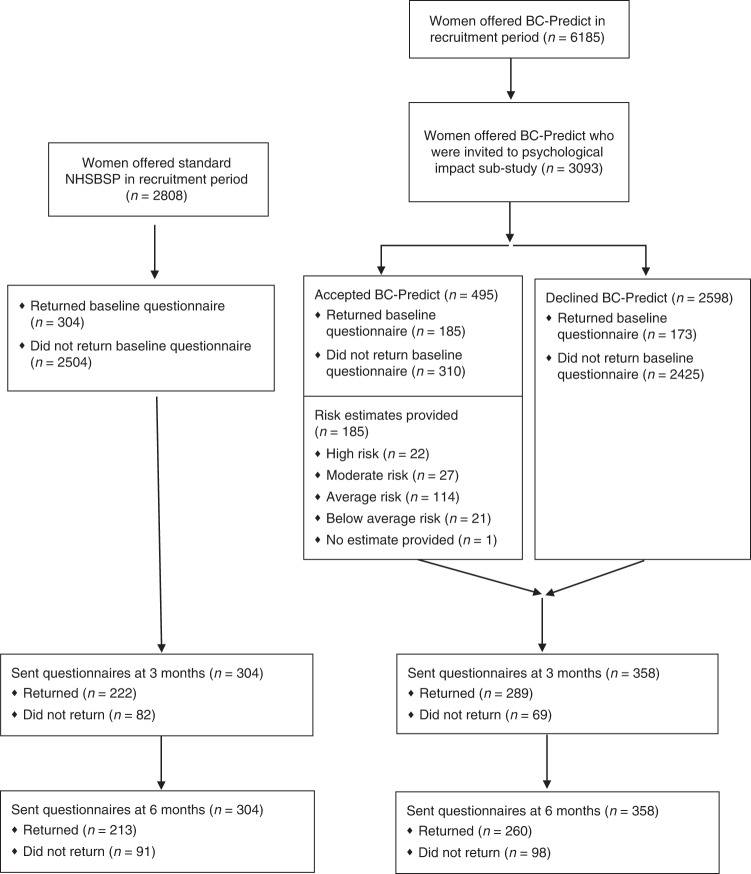
Flow diagram for the psychological impact sub-study of the BC-Predict study.

All women in the PROCAS2 study were invited from NHSBSP sites run by three services in North-West England, with women from five participating sites also being offered BC-Predict, and women from two other sites being offered only standard NHSBSP (with sites listed in Table 1). To ensure comparable numbers being invited during the recruitment period of the present nested questionnaire study, only half of women at the five sites offering BC-Predict were invited into the present questionnaire study (the first half of women on the daily list received from each site), whereas all women at the two sites offering only standard NHSBSP were invited. A flowchart shows the number of women included at each stage of the research (see Fig. 1).

**Table 1:** Demographic and clinical characteristics (mean [SD], % [*n*]) of women offered NHS Breast Screening Programme and BC-Predict (*n* = 662).

	Test groups		Test statistics	*P* values
	Offered NHS-BSP *n* = 304	Offered BC-Predict *n* = 358		
Age: mean (SD)	57.80 (4.44)	58.30 (4.22)	*t* (1, 660) = 0.433	*P* = 0.511
IMD: mean (SD)	5.18 (2.93)	7.08 (2.85)	*t* (1, 660) = 8.42	***P*** < **0.001**
1 (most deprived): % (*n*)	13.2% (40)	6.1% (22)		
2	12.5% (38)	4.2% (15)		
3	11.5% (35)	4.7% (17)		
4	5.6% (17)	6.7% (24)		
5	10.9% (33)	5.9% (21)		
6	7.2% (22)	7.0% (25)		
7	11.5% (35)	12.0% (43)		
8	10.5% (32)	10.6% (38)		
9	10.2% (31)	15.1% (54)		
10 (least deprived)	6.9% (21)	27.7% (99)		
Mammography: % (*n*)			*χ*^2^ (1) = 3.60	*P* = 0.058
(a) First (prevalent)	10.9% (33)	6.7% (24)		
(b) Repeat (incident)	89.1% (271)	93.3% (334)		
Mammography attendance: % (*n*)			*χ*^2^ (1) = 2.09	*P* = 0.148
(a) Attended appointment offered	94.7% (288)	91.9% (329)		
(b) Not attended this appointment	5.3% (16)	8.1% (29)		
Location: % (*n*)				
East Lancashire	0% (0)	19.8% (71)		
East Cheshire: Macclesfield	0% (0)	30.0% (107)		
East Cheshire: Stockport	0% (0)	12.3% (44)		
East Cheshire: Marple	0% (0)	14.1% (72)		
Greater Manchester: Oldham	0% (0)	20.1% (64)		
Greater Manchester: Hyde	55.3% (168)	0% (0)		
Greater Manchester: Salford	44.7% (136)	0% (0)		
(self-reported) ethnicity: (*n*)*				
(a) White		177		
(b) Asian or Asian British		0		
(c) Black or Black British		1		
(d) Mixed		1		
(e) Other		3		
(f) Prefer not to answer		2		
(g) Not indicated		174		
(self-reported) Jewish descent: (*n*)*		5		
Breast cancer diagnosis during the study	1	4		
Duration mammography appointment to join study (days): mean (SD)	17.52 (14.23)	15.48 (12.29)	*t* (1, 660) = 1.978	***P*** = **0.048**

^*^This information available for BC-Predict group only as based on self-report.

The bold values denote significant *P* values.

### Participants

Women who had mammograms scheduled at one of the seven participating sites in the 8-month recruitment period for this nested questionnaire study (November 2020 to July 2021) were eligible (although one site did not begin recruitment until February 2021). Two groups of women were invited to participate: (a) women invited for first-time screening at any age (“prevalent screens”), and (b) women invited during the screening round within which they would reach 60 years (“incident screens” i.e., women aged 57–63 years). Additional inclusion criteria were that the participant was born biologically female and was able to provide informed consent. Exclusion criteria were that the participant previously had breast cancer or had a bilateral mastectomy.

### Procedure

All women were invited to have a mammogram by the NHSBSP during the recruitment period. During the COVID-19 pandemic, the NHSBSP programme changed the breast screening invitation process to open-invite letters, and this continued for the duration of this study rather than reverting back to the previous usual practice of sending pre-allocated appointments.

Women offered BC-Predict were sent an additional invitation letter one to two working days after their breast screening invitation letter was sent. The BC-Predict invitation letter was sent along with the participant information sheet and instructions directing prospective participants to the online risk assessment platform. Following written informed consent, risk assessment was based on self-report questions completed by participants using the online risk assessment platform. Breast density estimates were automatically derived from mammography for the majority of women who agreed for images to be used. For some women, risk assessment also incorporated information from breast cancer single-nucleotide polymorphisms (SNPs), derived from DNA contained in saliva samples primarily collected via sampling kits sent and returned by post to the research office.

Women in the BC-Predict arm who took up the offer of BC-Predict and who received a clear mammogram result were sent a letter in the post providing them with their 10-year breast cancer risk ~6–8 weeks after their mammogram. The risk feedback letter informed women that they are at “high” (≥8% 10-year risk), “above average (moderate)” (≥5% but <8% 10-year risk), “average” (≥2% but <5% 10-year risk), or “below average” risk (<2% 10-year risk). Each letter explained how the risk estimates were derived, and the implications of these along with a leaflet providing additional detail on breast cancer risk factors, signs and symptoms of breast cancer and how risk might be managed. Full details of the development and refinement of risk letters and accompanying leaflets are described elsewhere [18], and examples of these are included as Appendices 1 and 2. Women at “high” or “above average (moderate)” risk were also encouraged to make an appointment at a Family History and Risk Prevention Clinic to talk about managing their risk.

From March 2021, the “below average” label was replaced with “low”, and women who received this result were also told that whether to extend the screening interval for women at this level of risk was being considered by the NHSBSP (this change was due to a planned within-study comparison of two ways of communicating information about below average/low risk that did not yield sufficient numbers of women for analyses to be worthwhile).

Women were asked to complete questionnaires at baseline, 3 months and 6 months after their initial mammogram appointment date, after providing explicit consent. For women in both BC-Predict and standard NHSBSP groups, the invitation to complete the questionnaire was sent approximately 7 days after their first offered mammogram appointment. The invite letter and patient information sheet provided interested women with instructions to complete an online consent form and questionnaire using a unique study identification number on SmartSurvey (https://www.smartsurvey.co.uk/). Paper copies were available on request. Women in both experimental groups only received follow-up questionnaire invites via letter once they had received a clear mammogram result and breast cancer status was negative. At both 3 months and 6 months, women who had not responded received a second follow-up questionnaire invite approximately two weeks later. Women who consented to the study but did not complete any baseline questions were not sent follow-up invites.

A fuller description of procedures for the larger PROCAS2 study and developmental work, which underpinned the refinement of procedures, technical processes and patient-facing material, is provided elsewhere [17].

### Measures

The following measures were administered:

*Perceived relative risk of developing breast cancer* was assessed with a single item that asked women to rate their risk of developing breast cancer in the next 10 years, compared with other women of their age, from “much lower”, “a bit lower”, “about the same”, “a bit higher” and “much higher” [19].

*State anxiety* was assessed using the six-item short form [20] of the Spielberger State-trait Anxiety Inventory [21], with participants responding to six emotion adjectives (e.g., “upset”) about their present feelings by selecting one of the following response options “not at all”, “somewhat”, “moderately” and “very much” (*α* = 0.86). A score of 49 on the full 20-item scale has been found in patients with a diagnosis of anxiety disorder [21]. In line with this, the present study dichotomised scores around the proportionate value of 14.7 on the six-item short scale to indicate clinical “cases” of anxiety.

*Breast cancer worry* was assessed using the Lerman Cancer Worry Scale [22], consisting of six statements such as: “how often have you thought about your chances of getting cancer?”. Participants endorsed one of the following response options “never”, “rarely”, “sometimes” and “almost all the time” (*α* = 0.87).

*Knowledge about breast cancer screening* was assessed using the 11 conceptual knowledge items taken from a standard measure [23] that has demonstrated sensitivity to an intervention to promote informed choices about screening (*α* = 0.30) [24].

*Attitudes towards screening* were assessed following a standard approach [25] with three items assessing whether women saw screening as good/ beneficial/important, with response options such as: “entirely good”, “mainly good”, “neither good nor bad”, “mainly bad” and “entirely bad” (*α* = 0.67).

*Intentions to attend future screening* also followed a standard approach [25] with a single item with the following response options: “strongly disagree”, “disagree”, “neither agree nor disagree”, “agree” and “strongly agree”.

*Satisfaction with the information* was assessed for women who received BC-Predict using four items from a previously published scale [26], which asked women how clear they found the information, how confusing they found it, how well informed they feel about their breast cancer risk, and how satisfied they are with the amount of information given. Response options were “strongly disagree”, “disagree”, “disagree somewhat”, “undecided”, “somewhat agree”, “agree” and “strongly agree (*α* = 0.90). These questions were included at 3 months and 6 months only.

*Demographic and clinical information* was obtained using information from the BC-Predict questionnaire that was used to estimate breast cancer risk for women in that experimental group. This was supplemented by core demographic and clinical information that were provided at the aggregate levels using a Confidentiality Advisory Group (CAG) approval for those women who declined BC-Predict and for women who were offered only standard NHSBSP and were therefore not asked to provide consent for the wider PROCAS2 study. *Area deprivation* was assessed for all women invited using the Index of Multiple Deprivation (IMD) deciles derived from postcodes of women invited, which indicated area deprivation for England in deciles from 1 (most deprived) to 10 (least deprived) [27].

### Data analysis

To assess differential response between allocated groups, comparisons of available demographic and clinical characteristics were made between: (a) women in the BC-Predict and standard NHSBSP groups who were invited to complete questionnaires, and (b) women who responded to questionnaires in the BC-Predict and standard NHSBSP groups. Analyses employed *t* tests for continuous variables and chi-squared analyses for categorical variables.

The main analyses focussed on comparing the responses of women offered BC-Predict with women offered standard NHSBSP at both follow-up timepoints. ANCOVA was used, with baseline responses to the same variables, age and IMD deciles as covariates. Analyses were conducted on all questionnaire measures at 3 months and 6 months, with the 6-month state anxiety measure being the a priori primary outcome for this study [17]. The a priori sample size calculation estimated that responses from *n* = 1054 women at 6 months (*n* = 527 in each of BC-Predict and standard NHSBSP groups) would yield 90% power to detect a small standardised mean difference of *d* = 0.2. This would be equivalent to a difference between adjacent response categories (e.g., “not at all” and “somewhat”) on 2.5 of the 20 items on the full form of the scale [17].

A similar approach was used to compare the responses of women in the BC-Predict group who accepted the offer of BC-Predict at follow-up, compared with women who declined this offer.

ANCOVA was also used to compare responses of the four groups of women in the BC-Predict group provided with different risk estimates (i.e., high, moderate, average and below average) at follow-up. These analyses for each variable controlled for baseline responses to the same variables with age and IMD deciles as covariates. Initial omnibus tests assessed whether differences in groups were statistically significant, and where these omnibus tests were significant, post hoc pairwise comparisons with all four groups were conducted.

Satisfaction with information received was compared between the four groups of women in the BC-Predict group provided with different risk estimates (i.e., high, moderate, average and below average) using ANOVA, as there was no baseline measure of this variable for the BC-Predict group and was not measured in the standard NHSBSP group.

All statistical tests were two-sided and used an a priori alpha level of 5%. A “completer only” analysis strategy was generally employed. However, given the dropout levels, the a priori primary outcome (comparison of 6-month outcome scores at six months between BC-Predict and standard NHSBSP groups) was repeated using a last occasion carried forward approach to missing data at 6 months, as a sensitivity analysis.

## Results

### Sample participation in relation to sample invited

In total, 5901 women were invited to participate in the present questionnaire study, of whom *n* = 3093 were offered BC-Predict and *n* = 2808 were offered NHSBSP. Of these 5901 women invited, *n* = 662 consented to the study (11.2%). Women were equally likely to participate if they were offered BC-Predict or NHSBSP (*χ*^2^ = 0.83, df=1, *N* = 5901, *P* = 0.363).

The age of women who participated (mean = 58.05 years, SD = 4.32) was not statistically different (*t* = 0.06, df = 5899, *P* = 0.949) from the age of women who did not participate (mean = 58.06 years, SD = 4.29). By contrast, women who participated had a higher IMD decile (*t* = 9.54, df = 5899, *P* < 0.001) (mean = 6.21, SD = 3.04) than women who did not participate (mean = 4.99, SD = 3.09).

### Comparability of the sample of women offered NHS Breast Screening Programme and BC-Predict

Of the *n* = 662 women who participated, *n* = 358 were offered BC-Predict and *n* = 304 were offered NHSBSP. Both groups were similar in terms of age, whether they attended mammography or not, or whether those screens were prevalent or incident (see Table 1). The women who participated in the BC-Predict group took a mean of 2 days fewer to return their questionnaires and had a higher IMD decile than women who participated in the NHSBSP group, and hence were less deprived (see Table 1). This is likely to have been due to the BC-Predict group being recruited partly from areas such as Macclesfield and Marple which are less deprived, relative to the areas where women offered NHSBSP were recruited from (see Table 1). Follow-up rates were reasonable at 3 months: 77.2% (BC-Predict group 80.7% and NHSBSP group 73.0%) and 6 months 71.5% (BC-Predict group 72.6% and NHSBSP group 70.1%).

### Comparisons of changes in outcome variables between women offered NHS Breast Screening Programme and BC-Predict

Comparisons of outcome scores for women offered BC-Predict and women offered NHSBSP produced no statistical significance differences on any outcome variable (see Table 2). This absence of difference between BC-Predict and NHSBSP groups applied at both 3 months and 6 months. A sensitivity analysis on 6-month state anxiety using a last occasion carried forward approach to missing data also identified no difference between BC-Predict and NHSBSP groups (see Appendix 3). It was notable that, in all analyses comparing changes in outcome variables according to allocated group, the strongest predictor was the baseline (t1) variable for the outcome variable of interest. For example, when the ANCOVA for state anxiety at 6 months was re-run as a linear regression to yield more readily interpretable estimates of association, baseline level of state anxiety was a very strong predictor (*β* = 0.58, *P* < 0.001).

**Table 2:** Self-report measures* (mean [SD]), at baseline and follow-up (3 months and 6 months post screening) with statistical tests to assess if differences in changes between women offered NHS Breast Screening Programme and BC-Predict (*N* = 662).

	Women offered NHS Breast Screening Programme			Women offered BC-Predict				
	Baseline (*n* = 304)	3 months (*n* = 222)	6 months (*n* = 213)	Baseline (*n* = 358)	3 months (*n* = 289)	6 months (*n* = 260)	Differences between groups at 3 months: test statistics (with *P* values)	Differences between groups at 6 months: test statistics (with *P* values)
Comparative risk perceptions	3.08 (0.75), *n* = 299	3.07 (0.75), *n* = 222	3.12 (0.75), *n* = 213	2.91 (0.80), *n* = 355	3.04 (0.92), *n* = 288	3.08 (0.95), *n* = 259	*F*(1, 502) = 1.207, *P* = 0.273	*F*(1, 464) = 1.019, *P* = 0.313
State anxiety	10.20 (3.80), *n* = 301	10.56 (3.84), *n* = 221	11.10 (4.19), *n* = 211	10.06 (3.67), *n* = 355	10.35 (3.81), *n* = 288	10.54 (3.77), *n* = 260	*F*(1, 502) = 0.017, *P* = 0.896	*F*(1, 462) = 1.314, *P* = 0.252
State anxiety (cases)^†^	46 (15.3%)	41 (18.6%)	42 (19.9%)	45 (12.7%)	39 (13.5%)	42 (16.2%)	*Χ*^2^ (1) = 1.01, *P* = 0.314	*Χ*^2^ (1) = 0.76, *P* = 0.384
Cancer worry	12.50 (3.20), *n* = 300	12.09 (2.97), *n* = 222	12.24 (3.03), *n* = 213	12.00 (3.02), *n* = 355	11.97 (2.94), *n* = 289	11.85 (3.04), *n* = 259	*F*(1, 504) = 1.467, *P* = 0.226	*F*(1, 465) = 0.418, *P* = 0.518
Screening knowledge	6.62 (1.44), *n* = 295		6.83 (1.53), *n* = 209	6.92 (1.57), *n* = 354		7.09 (1.55), *n* = 256		*F*(1, 456) = 1.207, *P* = 0.273
Attitudes towards screening	14.26 (1.37), *n* = 298		14.43 (1.09), *n* = 212	14.18 (1.35), *n* = 354		14.52 (1.00), *n* = 254		*F*(1, 458) = 1.049, *P* = 0.306
Intentions towards screening	4.50 (1.22), *n* = 298	4.74 (0.90), *n* = 222	4.54 (1.21), *n* = 213	4.45 (1.28), *n* = 355	4.68 (1.00), *n* = 288	4.55 (1.21), *n* = 259	*F*(1, 502) = 0.075, *n* = 0.785	*F*(1, 465) = 0.072, *P* = 0.788

^*^Higher scores indicate greater levels of each variable, i.e., higher perceived comparative risk, more state anxiety, more cancer worry, higher screening knowledge, more positive attitudes and higher levels of intentions.

^†^Logistic regression used rather than ANCOVA, as outcome variable dichotomous.

### Comparison of participants who accepted BC-Predict and participants who declined BC-Predict

The women who accepted the offer of BC-Predict did not significantly differ from the women who declined this offer on a number of variables, including age and state anxiety (see Table 3). However, they had slightly higher IMD deciles (hence less deprived), higher screening knowledge, lower risk perceptions and less worry about cancer.

**Table 3:** Self-report measures* (mean [SD]), with statistical tests to compare women who accepted BC-Predict and women who declined it (*n* = 358).

	Baseline Women accepted BC-Predict (*n* = 185)	Baseline Women declined BC-Predict (*n* = 173)	Differences between groups: test statistics (with *P* values)
Age	58.25 (4.29)	58.36 (4.15)	*t*(356) = 0.23, *P* = 0.816
IMD	7.55 (2.57)	6.58 (3.06)	***t***(**356)** **=** **3.23**, ***P*** = **0.001**
Duration of mammography appointment to join study (days)	15.87 (10.20)	15.06 (14.21)	*t*(356) = 0.63, *P* = 0.530
Comparative risk perceptions	2.82 (0.87)	3.02 (0.71)	***t***(**353)** **=** **2.39**, ***P*** = **0.017**
State anxiety	9.74 (3.56)	10.39 (3.77)	*t*(353) = 1.67, *P* = 0.097
Cancer worry	11.69 (2.83)	12.33 3.19)	***t***(**353)** **=** **2.01**, ***P*** = **0.045**
Screening knowledge	7.10 (1.64)	6.72 (1.46)	***t***(**352)** **=** **2.29**, ***P*** = **0.023**
Attitudes towards screening	14.21 (1.34)	14.16 (1.36)	*t*(352) = 0.33, *P* = 0.740
Intentions towards screening	4.43 (1.33)	**4.47** (**1.23)**	*t*(353) = 0.29, *P* = 0.775

^*^Higher scores indicate greater levels of each variable, i.e., higher perceived comparative risk, more state anxiety, more cancer worry, higher screening knowledge, more positive attitudes and higher levels of intentions.

The bold values denote significant *P* values.

### Comparison of changes in outcome variables according to risk estimate provided to women

There were several changes in outcome variables from baseline following receipt of risk estimates (see Table 4). First, the comparative risk perceptions changed in line with the risk estimates provided: risk perceptions increased in women told they were at higher risk and decreased in women told they were at lower risk. Post hoc tests revealed that the risk perceptions of all groups significantly differed at both follow-up timepoints, apart from the moderate and high-risk groups.

**Table 4:** Self-report measures* (mean [SD]), at baseline and follow-up (3 months and 6 months post screening) with statistical tests to assess if differences in changes according to risk estimate provided to women (*n* = 184).

		Baseline (*n* = 184)	3 months (*n* = 157)	6 months (*n* = 153)	Differences between groups at 3 months	Differences between groups at 6 months
Comparative risk perceptions	Total	2.82 (0.87), *n* = 184	3.05 (1.02), *n* = 157	3.07 (1.05), *n* = 153	***F***(3, 150) = **35.84,** ***P*** < **0.001**	***F***(3, 146) = **28.56,** ***P*** < **0.001**
	High	3.23 (1.02), *n* = 22	4.24 (0.77), *n* = 21	4.32 (0.75), *n* = 19		
	Moderate	3.15 (0.82), *n* = 27	3.95 (0.50), *n* = 21	3.86 (0.89), *n* = 22		
	Average	2.70 (0.86), *n* = 114	2.79 (0.77), *n* = 99	2.81 (0.74), *n* = 94		
	Below average	2.57 (0.60), *n* = 21	1.94 (0.93), *n* = 16	2.17 (1.20), *n* = 18		
State anxiety	Total	9.75 (3.56), *n* = 184	9.95 (3.58), *n* = 157	10.39 (3.75), *n* = 153	*F*(3, 150) = 0.94, *P* = 0.423	***F***(**3, 146)** **=** **3.73,** ***P*** = **0.013**
	High	9.00 (2.91), *n* = 22	9.00 (2.70), *n* = 21	9.84 (3.56), *n* = 19		
	Moderate	10.15 (4.29), *n* = 27	10.62 (4.50), *n* = 21	10.73 (3.72), *n* = 22		
	Average	9.62 (3.29), *n* = 114	9.82 (3.50), *n* = 99	9.80 (3.29), *n* = 94		
	Below average	10.76 (4.41), *n* = 21	11.13 (3.77), *n* = 16	13.67 (4.75), *n* = 18		
State anxiety (cases)^†^	Total	22 (12.0%)	15 (9.6%)	26 (17.0%)	Q(1) = 0.43, *N* = 157 *P* = 0.513	Q(1) = 2.90, *N* = 153 *P* = 0.088
	High	2 (9.1%)	1 (4.8%)	2 (10.5%)		
	Moderate	3 (11.1%)	4 (19.0%)	5 (22.7%)		
	Average	12 (10.5%)	8 (8.1%)	11 (11.7%)		
	Below average	5 (23.8%)	2 (12.5%)	8 (44.4%)		
Cancer worry	Total	11.69 (2.83), *n* = 184	11.84 (2.74), *n* = 157	11.54 (2.77), *n* = 153	*F*(3, 150) = 1.20, *P* = 0.311	***F***(**3,146)** **=** **4.15,** ***P*** = **0.008**
	High	12.77 (2.74), *n* = 22	13.10 (3.10), *n* = 21	13.21 (2.37), *n* = 19		
	Moderate	11.52 (2.86), *n* = 27	12.57 (2.42), *n* = 21	12.14 (2.75), *n* = 22		
	Average	11.76 (2.84), *n* = 114	11.65 (2.68), *n* = 99	11.35 (2.72), *n* = 94		
	Below average	10.38 (2.40), *n* = 21	10.44 (2.31), *n* = 16	10.06 (2.60), *n* = 18		
Screening knowledge	Total	7.10 (1.64), *n* = 184		7.18 (1.55), *n* = 153		*F*(3,146) = 0.39, *P* = 0.761
	High	7.09 (1.60), *n* = 22		7.42 (1.35), *n* = 19		
	Moderate	6.89 (1.72), *n* = 27		6.95 (1.81), *n* = 22		
	Average	7.11 (1.64), *n* = 114		7.15 (1.54), *n* = 94		
	Below average	7.33 (1.71), *n* = 21		7.33 (1.53), *n* = 18		
Attitudes towards screening	Total	14.21 (1.34), *n* = 184		14.59 (0.94), *n* = 150		***F***(3, 144) = **3.55,** ***P*** = **0.016**
	High	14.18 (1.33), *n* = 22		14.37 (1.12), *n* = 19		
	Moderate	14.15 (1.26), *n* = 27		14.90 (0.30), *n* = 21		
	Average	14.18 (1.41), *n* = 114		14.48 (1.05), *n* = 93		
	Below average	14.43 (1.08), *n* = 21		15.00 (0.00), *n* = 18		
Intentions towards screening	Total	4.43 (1.32), *n* = 184	4.68 (1.01), *n* = 157	4.62 (1.12), *n* = 153	*F*(3, 150) = 1.64, *P* = 0.182	*F*(3, 146) = 0.80, *P* = 0.497
	High	4.50 (1.19), *n* = 22	4.38 (1.43), *n* = 21	4.53 (1.26), *n* = 19		
	Moderate	4.78 (0.80), *n* = 27	5.00 (0.00), *n* = 21	4.95 (0.21), *n* = 22		
	Average	4.38 (1.39), *n* = 114	4.71 (0.93), *n* = 99	4.53 (1.24), *n* = 94		
	Below average	4.24 (1.61), *n* = 21	4.50 (1.37), *n* = 16	4.78 (0.94), *n* = 18		
Overall satisfaction with information^‡^	Total		25.39 (3.42), *n* = 151	25.67 (2.55), *n* = 147	*F*(3, 145) = 1.97, *P* = 0.122	*F*(3, 141) = 1.36, *P* = 0.258
	High		24.52 (3.14), *n* = 21	24.72 (2.49), *n* = 18		
	Moderate		24.20 (4.63), *n* = 21	25.40 (2.23), *n* = 20		
	Average		25.88 (2.46), *n* = 94	25.97 (2.36), *n* = 92		
	Below average		25.13 (5.85), *n* = 16	25.35 (3.71), *n* = 17		

^*^Higher scores indicate greater levels of each variable, i.e., higher perceived comparative risk, more state anxiety, more cancer worry, higher screening knowledge, more positive attitudes, and higher levels of intentions.

^†^Cochran Q test, for repeated dichotomous outcome variable across multiple test groups.

^‡^One-way ANOVA, as no baseline measure of satisfaction was obtained.

The bold values denote significant *P* values.

Cancer worry changed in line with the risk estimates provided at 6 months but not at 3 months, with women informed that they were at higher risk showing an increase in cancer worry and women informed that they were at lower risk showing a decrease in cancer worry. Post hoc tests revealed that cancer worry of all groups significantly differed at 6 months, apart from the moderate risk group who did not differ from the average and high-risk groups.

For state anxiety, there was a large increase in women told they were at below-average risk relative to other groups, which was apparent at 6 months but not at three months. Post hoc tests revealed that the women at below average had significantly higher state anxiety than all other groups at 6 months; no other group comparisons were significant.

There was little change apparent in screening knowledge or intentions to attend future screening, according to risk results received.

## Discussion

There was no evidence from this study that offering BC-Predict resulted in any effect on psychological harms, such as general anxiety or cancer worry, or benefits, such as screening knowledge, compared to offering NHSBSP. There was a clear pattern of the psychological impact of receiving different risk estimates provided by BC-Predict. There was a general acceptance of personal risk estimates, where women told they were at higher risk-rated their own risk as higher than baseline, and women told they were at lower risk that rated their own risk as lower. Similarly, there was an increase in worry about cancer in women told they were at higher risk and a decrease in worry about cancer in women told they were at lower risk. The changes were generally not large however, and there was no evidence of increases in clinical levels of anxiety in the BC-Predict group relative to the NHSBSP group. It was also notable that the strongest predictor of general anxiety and cancer worry at follow-up was baseline levels of these same variables, i.e., those women who experienced psychological distress at follow-up were the same women who experienced psychological distress at baseline, irrespective of whether they received BC-Predict or not, or any particular risk estimate.

The present study has several strengths in comparison with previous research. Most notably, unlike in previous studies of the psychological impact of receiving risk-stratified screening in the PROCAS study [11, 28], in this study, women received risk estimates within a few weeks of providing risk information and receiving a negative screening test result. This is in line with what should happen were risk-stratified screening to be introduced as part of routine breast cancer screening. Second, this study compared the reactions of women offered BC-Predict or standard NHSBSP, rather than just those who took up the offer of BC-Predict or NHSBSP, as this is the most appropriate comparison to establish any potential psychological harms. Third, the use of a comparison group of women undergoing NHSBSP at the same time is the most appropriate to establishing which changes were due to receiving risk estimates and additional screening or prevention offers, and which were due to just undergoing screening, which is itself associated with changes in anxiety [29]. Importantly, the use of baseline measures clearly showed that there were comparatively small changes in general anxiety and cancer worry brought about by the offers of BC-Predict and receipt of subsequent risk estimates. By contrast, large amounts of variance in follow-up measures were explained by baseline measures of the same variable. This showed that women who were distressed at follow-up were distressed at baseline.

The main limitation of the present research was that we did not randomise women to receive either BC-Predict or standard NHSBSP, which should have resulted in comparable groups. By contrast, due to the approach taken the comparison group was not well matched on deprivation indices with the BC-Predict group. This lack of matching was partly due to a change in design from that originally planned, which we considered to be the best design that was feasible, given the pragmatic challenges that randomisation would have presented in this study [17]. Our original plan was for attendees at NHSBSP in each location to be offered BC-Predict for a period of 8 months and NHSBSP for a further period of 8 months, with order counter-balanced within sites. However, this was not possible due to COVID-19 affecting the NHSBSP, which was suspended prior to the data collection period in this study, and then operating at reduced capacity during the data collection period. It should be noted that there was a good spread of women in all 10 IMD deciles across both the BC-Predict and NHSBSP groups, albeit with the least deprived deciles of women overrepresented in the BC-Predict group. Importantly, all analyses reported controlled for IMD deciles, which was not strongly related to the outcome variables.

The other major limitation with the present research was the 11.2% questionnaire response rate of women invited to participate in the present questionnaire study. This rate was attributable to multiple factors, including lower uptake to NHSBSP during this period, as women were required to actively make an appointment rather than be allocated an appointment. It is notable that the uptake to NHSBSP in the present areas during the period of this study was only 60.7%, and that uptake of BC-Predict amongst women offered it was only 16.1% [30]. Thus although the response rate of the present study compared unfavourably to the 36% questionnaire response rate of the first PROCAS study, in this previous study, this response rate was already from a selected sample, as women in this previous study had previously agreed to participate in screening and consented to the wider PROCAS study. Thus, although the low response rate increases the risk of response bias, the approach in this study of inviting everyone who was offered BC-Predict or standard NHSBSP did not involve the selection biases of previous studies. It should be noted that women were equally likely to participate if they were offered BC-Predict or NHSBSP, which suggests that there was no evidence of differential response biases, which is probably a more important threat to validity of conclusions than low response rate per se.

A third potential limitation is that the number of women recruited was substantially lower than originally intended, at least partly due to the lower-than-anticipated response rate just mentioned. The sample size calculations indicated that *n* = 1054 women would be sufficient to detect a small effect on state anxiety at 6 months, whereas only 662 women were recruited, with 473 retained at 6 months follow-up. However, even though the present study had a smaller sample size than anticipated, there was little evidence of any differential effects of BC-Predict compared to standard NHSBSP for any of the variables listed in Table 2.

The present research provides no evidence to support the idea that BC-Predict resulted in any psychological distress compared to the offer of standard NHSBSP. This finding is in accord with findings from the first PROCAS study [13], despite quite different methods used in the two studies, which have contrasting strengths and weaknesses. This should increase confidence in the absence of harms found. Further, although one should generally be cautious about interpreting null findings as indicating no difference, there were (non-significantly) higher levels of cancer worry and general anxiety in the NHSBSP group than the BC-Predict at 6 months.

An unexpected finding was the increase in state anxiety in women who were told that they were at low risk. This finding was unexpected for three reasons. First, previous research has found that women who were told they were at lower risk showed a reduction in state anxiety [13]. Second, this increase was apparent at 6 months but not at three months, but the women received no additional intervention content or screening healthcare contact between three and 6 months. Third, in the present study, these same low-risk women showed a reduction in cancer worry over the same period. This indicates that whatever was causing the increase in general anxiety, it was not concerned about cancer.

This study also found no support for any impact on future intentions to change screening, of either offering BC-Predict or of receiving any particular risk estimate. This finding is again in accordance with intentions to change behaviour found in the first PROCAS study [13]. In addition, a cohort study of over 26,000 women who participated in the first PROCAS found no support for the idea that screening attendance would be reduced in either women at either higher risk (due to avoidant behaviour) or lower risk (due to false reassurance) [31]. This is in line with the wider literature on receiving personalised risk information producing little influence on behaviour [32].

The final notable finding is the lack of effect of participating in BC-Predict on cancer screening knowledge. It has been proposed that one of the potential benefits of risk-stratified screening is an increase in understanding of screening [9]. Given that knowledge is a central component of informed decision-making [15], this is a potentially important benefit. There is a dearth of evidence on this point [16], which the present study addresses using a measure that has previously demonstrated validity [23]. The lack of effect of BC-Predict or risk estimates provided on screening knowledge suggests that the offer of risk-stratified screening alone does not stimulate a wider consideration of screening issues such as false negative screening test results or overdiagnosis.

Taken as a whole, the key implication of this study is to provide the best available evidence that risk-stratified screening is unlikely to produce psychological harms in the women that are offered it. This finding is in line with previous research, but the present study has a number of strengths, including more appropriate timing of questionnaire distribution in relation to participation in risk-stratified screening, more appropriate control groups and inclusion of high-risk women [17]. It is also notable that in the present study, the influence of risk estimates communicated on women’s ratings of personal risk indicates that the risk information provided was understood, but this understanding is not intrinsically problematic. These findings are therefore in line with the wider empirical literature which consistently shows a lack of impact of personalised risk information [32–34], despite the apparent beliefs of many researchers who are resistant to this consistent finding.

The present findings add further to the weight of evidence showing no major psychological impact of risk-stratified screening causes psychological harms in the NHSBSP. It is possible that risk-stratified screening in screening programmes in other countries or when delivered in other ways may cause psychological harms, and the ongoing MyPeBS study should address this possibility, as well as consider any harms or benefits for up to four years post screening [35]. The present study will be able to provide further information of the impact of risk-stratified screening on informed choices when the women included in the present study actually attend or do not attend future rounds of screening, and relate this to their knowledge and attitudes to screening reported in the present paper. There are also other potential harms and benefits of risk-stratified screening that the wider PROCAS2 study has generated data on [30], and will be able to report on shortly, in line with the pre-registered objectives [17].

In sum, this study aimed to identify any psychological harms of risk-stratified screening brought about by BC-Predict, as this is one of the major barriers to risk-stratified screening that has been identified [10]. We believe this study is the best-designed study to address this question that has been carried out to date, and notwithstanding study limitations, the results are fairly unambiguous: risk-stratified screening does not appear to cause any major psychological harms, and this should not be a barrier to implementation.

## Supplementary information

legends for supplementary files

Appendix 1: example BC-Predict feedback letter (for average risk woman)

Appendix 2: example BC-Predict results leaflet

Appendix 3: sensitivity analysis

## Data Availability

Available on request from the first author.
