# Supplementary material for: Psychological impact of risk-stratified screening as part of the NHS Breast Screening Programme: multi-site non-randomised comparison of BC-Predict versus usual screening (NCT04359420)
Source: Br J Cancer. 2023 Feb 11;128(8):1548–58. doi: 10.1038/s41416-023-02156-7 (PMC9922101; doi:10.1038/s41416-023-02156-7)
Supplement: Supplementary file 1 — legends for supplementary files [file 41416_2023_2156_MOESM1_ESM.docx]

**Supplementary material legends**

1. **Example risk feedback letter used in BC-Predict**
2. **Example risk feedback leaflet used in BC-Predict**
3. **Sensitivity analysis of self-report measures* (mean [SD]), at baseline and follow-up with last occasion carried forward imputation (three months and six months post-screening) w****ith statistical tests to assess if differences in changes between women offered NHS Breast Screening Programme and BC-Predict (N=660)**
