# Supplementary material for: Psychological impact of risk-stratified screening as part of the NHS Breast Screening Programme: multi-site non-randomised comparison of BC-Predict versus usual screening (NCT04359420)
Source: Br J Cancer. 2023 Feb 11;128(8):1548–58. doi: 10.1038/s41416-023-02156-7 (PMC9922101; doi:10.1038/s41416-023-02156-7)
Supplement: Supplementary file 2 — Appendix 1: example BC-Predict feedback letter (for average risk woman) [file 41416_2023_2156_MOESM2_ESM.doc]

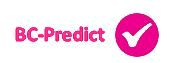
**INSERT TRUST LOGO**

Ref: 1234567895/00000

**INSERT TRUST ADDRESS**

**INSERT NAME INSERT ADDRESS INSERT ADDRESS INSERT ADDRESS**

**INSERT POSTCODE**

Date: **INSERT DATE** Tel: +44 (0)161 291 4408

Email: BCPredict@mft.nhs.uk

Dear Ms Tester,

**RE: Breast Cancer (BC)-Predict Study**

**IMPORTANT: ALL CLEAR after your recent mammogram. This is NOT a recall.**

Thank-you for taking part in the BC-Predict study. As part of that study we asked you to complete a questionnaire before you attended your mammogram. In this questionnaire you gave us information which enabled us to work out your risk of developing breast cancer in the next 10 years. Your risk is calculated from a combination of factors associated with your family history, lifestyle and breast density (the amount of tissue in your breast that is not fat).

Your risk of developing breast cancer in the next 10 years was calculated to be **average risk.** This means that 2 - 4% of women in your risk category will develop breast cancer **within the next 10 years.**

The table below provides you with information about your risk category and where you are in relation to other women:

**High**

**80 to 92% of women will NOT develop breast cancer.**

8 to 20% of these women will develop the disease.

**YOUR RISK**

**Above average (moderate)**

**93 to 95% of women will NOT develop breast cancer.**

5 to 7% of these women will develop the disease.

**Below average**

**99% of women will NOT develop breast cancer.**

0 to 1% of women will develop the disease.

**Average**

**96 to 98% of women will NOT develop breast cancer.**

2 to 4% of these women will develop the disease.

**High**

**80 to 92% of women will NOT develop breast cancer.**

8 to 20% of these women will develop the disease.

**YOUR RISK**

**Above average (moderate)**

**93 to 95% of women will NOT develop breast cancer.**

5 to 7% of these women will develop the disease.

**Below average**

**99% of women will NOT develop breast cancer.**

0 to 1% of women will develop the disease.

**Average**

**96 to 98% of women will NOT develop breast cancer.**

2 to 4% of these women will develop the disease.

**High**

**80 to 92% of women will NOT develop breast cancer.**

8 to 20% of these women will develop the disease.

**YOUR RISK**

**Above average (moderate)**

**93 to 95% of women will NOT develop breast cancer.**

5 to 7% of these women will develop the disease.

**Below average**

**99% of women will NOT develop breast cancer.**

0 to 1% of women will develop the disease.

**Average**

**96 to 98% of women will NOT develop breast cancer.**

2 to 4% of these women will develop the disease.

**High**

**80 to 92% of women will NOT develop breast cancer.**

8 to 20% of these women will develop the disease.

**YOUR RISK**

**Above average (moderate)**

**93 to 95% of women will NOT develop breast cancer.**

5 to 7% of these women will develop the disease.

**Below average**

**99% of women will NOT develop breast cancer.**

0 to 1% of women will develop the disease.

**Average**

**96 to 98% of women will NOT develop breast cancer.**

2 to 4% of these women will develop the disease.

**High**

**80 to 92% of women will NOT develop breast cancer.**

8 to 20% of these women will develop the disease.

**YOUR RISK**

**Above average (moderate)**

**93 to 95% of women will NOT develop breast cancer.**

5 to 7% of these women will develop the disease.

**Low**

**More than 98% of women will NOT develop breast cancer.**

Less than 2% of women will develop the disease.

**Average**

**96 to 98% of women will NOT develop breast cancer.**

2 to 4% of these women will develop the disease.

Based on your questionnaire answers and mammogram, the following may have increased your risk of breast cancer:


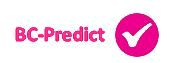
**INSERT TRUST LOGO**

- **[INSERT RISK FACTOR 1]**
- **[INSERT RISK FACTOR 2]**
- **[INSERT RISK FACTOR 3]**

However, these factor(s) may have helped to prevent your risk from being any higher:

- **[INSERT PREVENTATIVE FACTOR 1]**
- **[INSERT PREVENTATIVE FACTOR 2]**
- **[INSERT PREVENTATIVE FACTOR 3]**

Your risk of breast cancer can be reduced by up to 25% by making positive lifestyle changes, such as; adopting a healthy diet, taking regular exercise and losing weight (if needed). Such changes can also help reduce your risk of developing other diseases, such as heart disease, diabetes and dementia. More information on the ways to reduce your risk, together with the signs and symptoms of breast cancer are provided in the accompanying leaflet.

Please remember that even though you have an increased risk of developing breast cancer in the next 10 years, **96 to 98%** **of women in your risk group will NOT develop breast cancer.**

Should you have any questions, or wish to discuss your risk information with a Clinician, please get in touch with the BC-Predict Study team on **0161 291 4408**, Monday to Friday 10am to 2pm.

Yours sincerely,


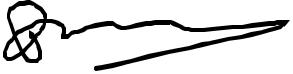


Prof D. Gareth Evans

**Queries about your breast screening appointments**

If you have any questions about your routine breast screening appointments, please call **[INSERT LOCAL SCREENING** **OFFICE NUMBER]**, or email: **[LOCAL SCREENING OFFICE EMAIL].**
