# Supplementary material for: Psychological impact of risk-stratified screening as part of the NHS Breast Screening Programme: multi-site non-randomised comparison of BC-Predict versus usual screening (NCT04359420)
Source: Br J Cancer. 2023 Feb 11;128(8):1548–58. doi: 10.1038/s41416-023-02156-7 (PMC9922101; doi:10.1038/s41416-023-02156-7)
Supplement: Supplementary file 3 — Appendix 2: example BC-Predict results leaflet [file 41416_2023_2156_MOESM3_ESM.pdf]

The University of Manchester

Manchester University NHS Foundation Trust

BC-Predict

Breast cancer risk factors

Reducing your risk

Signs and symptoms of breast cancer

This leaflet is designed to accompany your risk feedback letter

The Breast Cancer (BC) Predict Study

You took part in a research study run by the Prevent Breast Cancer Research Unit and Nightingale Centre at Manchester University NHS Foundation Trust (MFT). This study is called BC-Predict.

BC-Predict aims to assess whether providing women with their estimated risk of developing breast cancer is feasible within the current NHS Breast Screening Programme. For this, we assessed your risk of developing breast cancer in the next 10 years.

Background to breast cancer

Breast cancer is the most common type of cancer in the UK.

In 2016, 55,122 women were diagnosed with breast cancer from the age of 20. Of these, 78% of women (3 out of 4) are predicted to live for 10 or more years after diagnosis.

This high rate of survival is in part due to cancers being detected at an earlier stage. The earlier a cancer is detected, the more effectively it can be treated.

Breast cancer risk factors

Many factors contribute to your risk of developing breast cancer. Some of these are possible to change (modifiable) and some are not possible to change (non-modifiable):

Modifiable risk factors:

- Being overweight
- Drinking more than 14 units of alcohol a week – such as 7 small glasses of wine
- Being physically inactive – such as doing less than 2.5 hours of exercise a week
- Smoking

Non-modifiable risk factors:

- Age
- Family history
- Breast density
- Never having had children
- Being 30 years or older at the birth of your first child
- Starting your periods before the age of 12
- Going through the menopause after the age of 55

More detail about these factors can be found on the following page. Your risk has been calculated based on these factors.

Non-Modifiable risk factors

Age

The older you are the greater your chances of developing breast cancer. Approximately 4 out of 5 breast cancers occur in women aged 50 and over.

Family history

Women who have relatives diagnosed with breast cancer have a higher risk of developing the disease.

• Risk increases with the number of close relatives diagnosed, especially if they were diagnosed at a younger age.

•Studies show that more than 8 out of 10 breast cancers occur in women with no close relatives diagnosed with the disease.

Breast density

Breasts are made up of glandular tissue, connective tissue and fat. Women with dense breasts have more glandular and connective tissue than fat. The risk of breast cancer is higher in women with dense breasts because there is more tissue that could potentially become cancerous. Dense breast tissue can make a mammogram more difficult to read because abnormal lumps are harder to detect. See more info here: https://preventbreastcancer.org.uk/why-breast-density-matters/

Hormones and reproduction

The female sex hormones, oestrogen and progesterone, can affect the development of breast cancer in the following ways:

• Hormone Replacement Therapy (HRT) increases the risk of breast cancer. The risk associated with HRT is reduced as soon as you stop taking it but remains a little raised for 5 years.

• Oral contraceptives (also known as the Pill) increase the risk of breast cancer but can reduce the risk of ovarian and womb cancers. The risk associated with the Pill is reduced 10 years after you stop taking it.

• Starting your periods at a younger age or having a late menopause increases the risk of breast cancer. This is due to being exposed to the female sex hormones for longer.

• Having children below the age of 30 and breast feeding both lower the chances of developing the disease.

Risk factors you can change

Maintain a healthy weight

Being overweight and gaining weight throughout adult life increases the risk of developing breast cancer, especially after the menopause. Try to maintain a healthy weight by combining a balanced diet, including plenty of fruit and vegetables. For advice go to: www.nhs.uk/live-well/eat-well/

Physical activity

Women who are active are less likely to develop breast cancer than non-active women. Try to do at least 2.5 hours of moderate physical activity a week, such as 30 minutes of brisk walking five times a week and strength exercises. For advice, consult your GP practice or go to NHS Choices: www.nhs.uk/live-well/exercise/free-fitness-ideas/

Limiting alcohol

Drinking alcohol raises the risk of breast cancer. Try to keep to sensible intakes of less than 14 units a week, or 2-3 units a day ensuring you have at least 2 alcohol free days a week.

- 14 units is around a bottle and a half of wine (7 small glasses) a week or 10 measures of spirit a week.
- 2-3 units a day of alcohol is approximately 1 medium-large glass of wine, a pint of beer or 2 measures of spirits.

For advice go to : www.nhs.uk/live-well/alcohol-support/

Not smoking

As well as increasing your risk of developing heart disease and lung cancer, smoking increases your risk of developing breast cancer. For advice on how to stop smoking, contact your GP or join an NHS Stop Smoking Service. You can find your local service here www.nhs.uk/smokefree or call the Smokefree National Helpline to speak to a trained adviser on 0800 0224 332.

Preventative medication

Together with maintaining a healthy lifestyle, women at **above average (moderate)** and **high risk** may also be eligible to take a drug for prevention, please see your accompanying risk letter for more details.

Signs and symptoms of breast cancer

If you get to know how your breasts normally look and feel, you will be more likely to spot any changes that could be signs of breast cancer. This is important, even if you have been for breast screening. Look out for the following:

- A lump or swelling in the breast
- A change in the nipple. The nipple might be pulled back into the breast, or change shape. You might have a rash that makes the nipple look red and scaly, or have blood or another fluid coming from the nipple.
- A change in how your breasts feel or look. They may feel heavy or uneven. The skin may look dimpled, red or hot to touch. The size and shape of the breast may change.
- Pain or discomfort in the breast or armpit.
- A swelling or lump in the armpit.

For more information, go here: https://preventbreastcancer.org.uk/about-breast-cancer/signs-symptoms/

Even women with low risk can still develop breast cancer, so it is important to know the signs of breast cancer and what you can do to reduce your risk.

Talking about and sharing any concerns you may have about your risk of developing breast cancer with your loved ones may help to reduce some of your worries.

If you have any changes to your breasts, you should make an appointment to see your GP straight away. **You should tell the GP practice why when making an appointment.** It is unlikely that you have breast cancer, but if you do, being diagnosed and treated at an early stage makes it more likely that you can be successfully treated.

If you have a family history of breast cancer you should discuss this with your GP who may refer you to a Family History Clinic (FHC) where you can discuss your risk further.

If you have any questions about your routine breast screening appointments, you can contact your GP or your local breast screening service. Please find the contact details at the end of your risk letter.

You may find the following websites helpful to learn more about breast cancer and ways to reduce your risk:

Prevent Breast Cancer:
www.preventbreastcancer.org.uk
Cancer Research UK: www.cancerresearchuk.org
Macmillan Cancer Support: www.macmillan.org.uk
Breast Cancer Care UK: www.breastcancercare.org.uk
Breast Cancer Now: www.breastcancernow.org

For further details regarding the BC-Predict study, please contact:

BC-Predict team:
Prevent Breast Cancer Research Unit at the **Nightingale Breast Screening Centre**
Manchester University NHS Foundation Trust
Wythenshawe Hospital
Southmoor road,
Manchester
M23 9LT

Telephone: 0161 291 4408
E-mail: BCPredict@mft.nhs.uk

BC-Predict risk feedback leaflet: v2.1 1st February 2021
Review date latest: November 2020
