# Supplementary material for: Psychological impact of risk-stratified screening as part of the NHS Breast Screening Programme: multi-site non-randomised comparison of BC-Predict versus usual screening (NCT04359420)
Source: Br J Cancer. 2023 Feb 11;128(8):1548–58. doi: 10.1038/s41416-023-02156-7 (PMC9922101; doi:10.1038/s41416-023-02156-7)
Supplement: Supplementary file 4 — Appendix 3: sensitivity analysis [file 41416_2023_2156_MOESM4_ESM.docx]

**Sensitivity analysis of self-report measures* (mean [SD]), at baseline and follow-up with last occasion carried forward imputation (three months and six months post-screening) w****ith statistical tests to assess if differences in changes between women offered NHS Breast Screening Programme and BC-Predict (N=660)**

|  | Women offered NHS Breast Screening Programme (n=303) | | | Women offered BC-Predict (n=357) | | |  | |
| --- | --- | --- | --- | --- | --- | --- | --- | --- |
|  | BASELINE | THREE MONTHS | SIX MONTHS | BASELINE | THREE MONTHS | SIX MONTHS | Differences between groups at THREE MONTHS: test statistics (with p-values) | Differences between groups at SIX MONTHS: test statistics (with p-values) |
| **Comparative risk perceptions** | 3.08 (0.75) n=299 | 3.10 (0.75) n=300 | 3.14 (0.75) n=301 | 2.91 (0.80) n=355 | 3.04 (0.88) n=357 | 3.10 (0.92) n=357 | F(1,649)=1.414 p=0.235 | F(1,649)=1.508 p=0.220 |
| **State anxiety** | 10.20 (3.80)  N=301 | 10.58 (3.79)  N=301 | 11.08 (11.08) n=303 | 10.06 (3.67)  N=355 | 10.44 (3.82) n=357 | 10.52 (3.68) n=357 | F(1,651)=0.028 p=0.866 | F(1,651)=1.031 p=0.310 |
| **Cancer worry** | 12.50 (3.20) n=300 | 12.37 (3.12) n=300 | 12.42 (3.09) n=301 | 12.00 (3.02) n=355 | 12.03 (2.94) n=357 | 11.98 (3.09) n=357 | F(1,650)=1.219 p=0.270 | F(1,650)=0.018 p=0.893 |
| **Screening knowledge** | 6.62 (1.44) n=295 |  | 6.72 (1.53) n=297 | 6.92 (1.57) n=354 |  | 6.94 (1.50) n=356 |  | F(1,644)=0.456 p=0.500 |
| **Attitudes towards screening** | 14.26 (1.37) n=298 |  | 14.37 (1.29) n=300 | 14.18 (1.35) n=354 |  | 14.36 (1.17) n=355 |  | F(1,647)=0.317 p=0.573 |
| **Intentions towards screening** | 4.50 (1.22) n=298 | 4.65 (1.02) n=299 | 4.55 (1.18) n=300 | 4.45 (1.28) n=355 | 4.68 (1.00) n=357 | 4.55 (1.19) n=357 | F(1,648)=0.577 p=0.448 | F(1,648)=0.171 p=0.679 |

* Higher scores indicate greater levels of each variable, i.e. higher perceived comparative risk, more state anxiety, more cancer worry, higher screening knowledge, more positive attitudes, and higher levels of intentions.
